# Supplementary material for: Complement factor H protects tumor cell-derived exosomes from complement-dependent lysis and phagocytosis
Source: PLoS One. 2021 Jun 16;16(6):e0252577. doi: 10.1371/journal.pone.0252577 (PMC8208531; doi:10.1371/journal.pone.0252577)
Supplement: S1 Raw images — (PDF) [file pone.0252577.s002.pdf]

Fig 1 original GT103 western blot

Fig 1 original anti-CD63 western blot

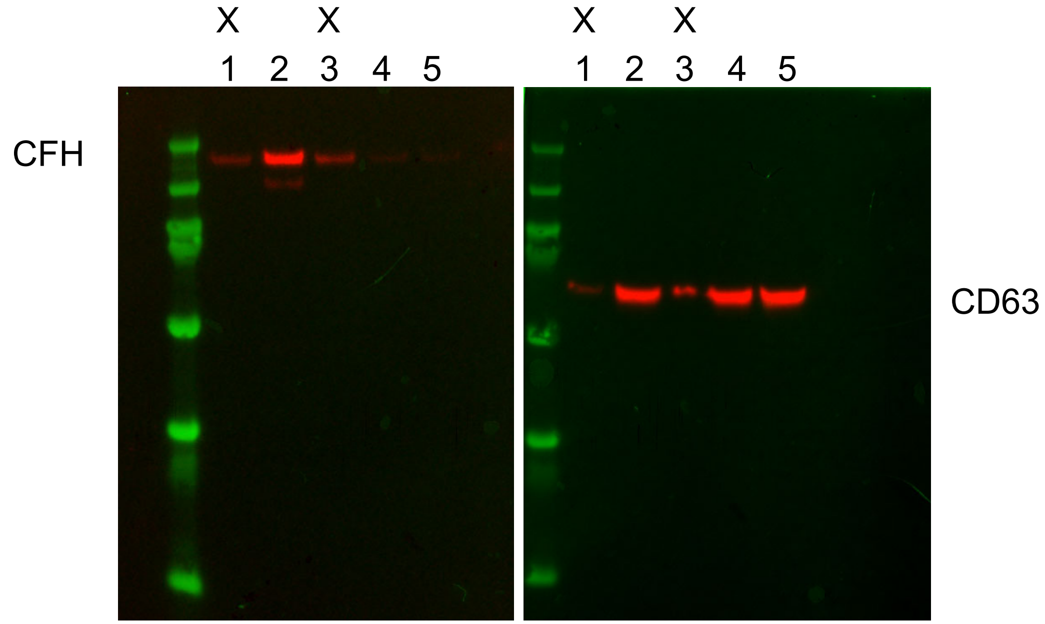

Lanes included in the final composite **Fig 1** are lanes 2, 4, 5 on the original blots.

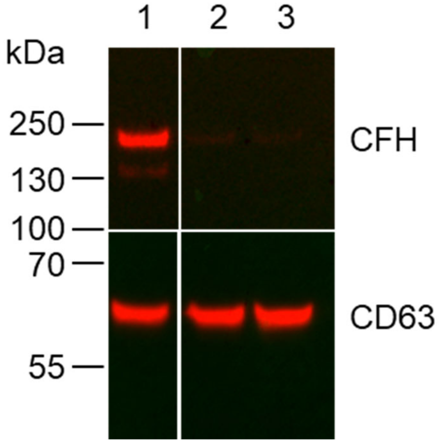

Creation of Fig 1: EVs from wild type and two CFH knockout mutant CMT167 cell lines were run on a gel and western blotted. This single western blot was sequentially probed with antibodies against CFH (GT103) and CD63, stripping the blot in between. Lanes 1, 2, and 3 contain 0.5 µg, 7.5 µg, and 2 µg EV protein, respectively from wild type CMT167; lanes 4 and 5 contain 7.5 µg from two different CFH-CRISPR/Cas9 knockout cell lines. The molecular masses of the protein markers are: 250, 130, 100, 70, 55, 35, 25, and 15 kDa.

The chemiluminescence on the blot as a result of the HRP reaction was captured by a ChemiDoc imager (BioRad). The imager also took a photographic image of the prestained protein markers and combined the two images into a single output. To create **Fig 1**, we merged the GT103 probed blot with the anti-CD63 probed blot at approximately the 100 kDa marker into a composite image that was edited to include only lanes containing 7.5 µg of EV protein per lane in the final figure (lanes 2, 4, and 5 in the originals).

Lanes in the final figure contain 7.5 mg EV proteir from 1, wild type CMT167; 2 and 3, two different CFH-CRISPR /Cas9 knockout cell lines.

Fig 2A original GT103 western blot

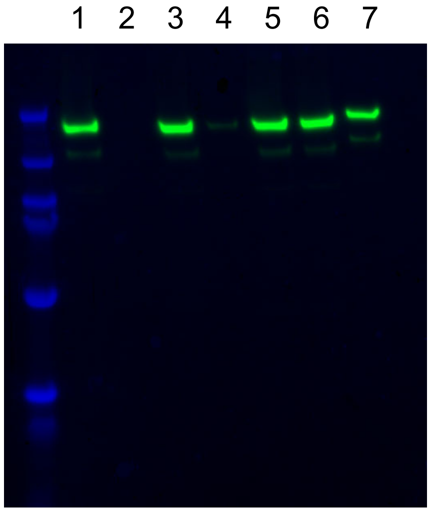

Fig 2A original anti-CD63 western blot

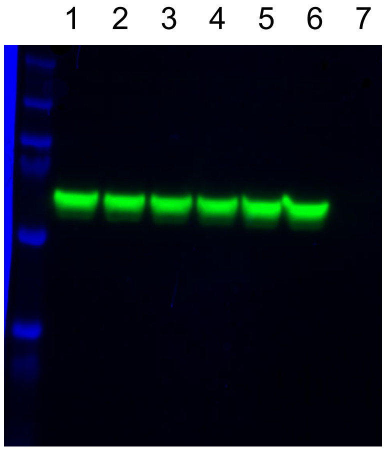

The final composite **Fig 2A**

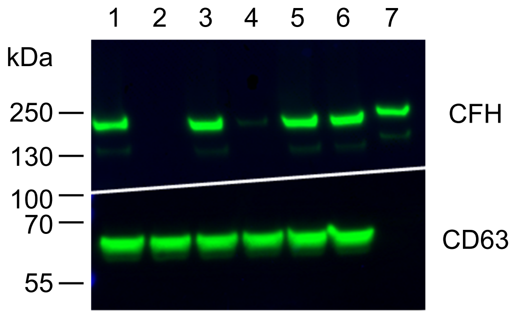

Creation of Fig 2A: EVs from 6 cell lines were run on a gel along with a CFH standard and western blotted. This single western blot was sequentially probed with antibodies against CFH (GT103) and CD63, stripping the blot in between. Lanes contain EV protein from the following cell lines: 1, B16F10; 2, B16; 3, LLC-met; 4, LLC 5, A549; 6, NCI-H460. Lane 7 contains 25 ng purified human CFH (Complement Technology, Inc., Tyler, TX). The molecular masses of the protein markers are: 250, 130, 100, 70, 55, 35, 25, and 15 kDa.

The chemiluminescence on the blot as a result of the HRP reaction was captured by a ChemiDoc imager (BioRad). The imager also took a photographic image of the prestained protein markers and combined the two images into a single output. To create **Fig 2A**, we merged the GT103 probed blot with the anti-CD63 probed blot at approximately the 100 kDa marker, into a composite image. (The CD63 image had to be rotated in order to align the lanes.)

Fig. 3 original western blot of lung cancer patient *extracellular vesicles* (ultracentrifugation pellets) probed with GT103

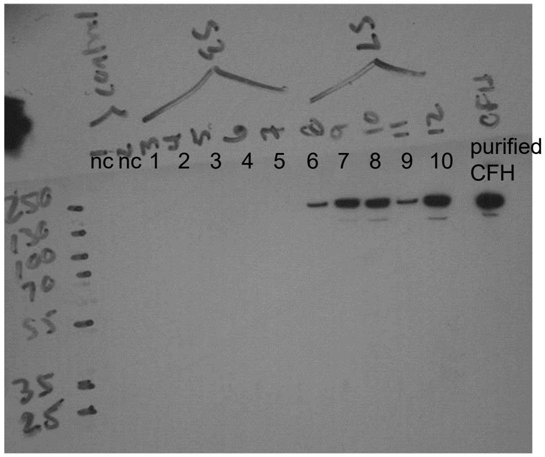

Fig. 3 original western blot of lung cancer patient *plasma* probed with GT103

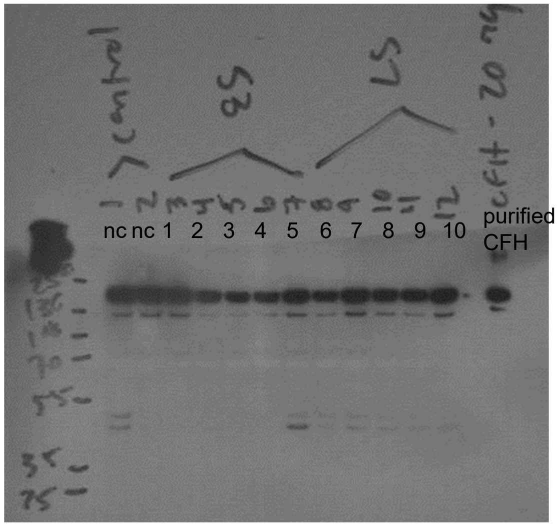

Key

1 – Control with no cancer (nc)
2 – Control with no cancer (nc)
1 – Squamous cell carcinoma stage IB
2 – Adenocarcinoma stage IB
3 – Adenocarcinoma stage IA
4 – Adenocarcinoma stage IB
5 – Squamous cell lung cancer stage IB
6 – Squamous cell carcinoma stage IIIA
7 – Adenocarcinoma stage IIIA
8 – Small cell lung cancer stage IV
9 – Squamous cell carcinoma stage III
10 – Adenocarcinoma stage IIIA
CFH Protein, human, 20 ng (Complement Technology, Inc.)

Creation of Fig 3: This figure was made from two independent blots each probed with GT103 antibody. Extracellular vesicles were isolated from patient plasma by ultracentrifugation as described in the Methods. Plasma from the same patients were run on a second gel. Gels were western blotted and probed with a murinized IgG2A version of GT103 as primary antibody and anti-mouse-HRP as secondary antibody-conjugate. The first two lanes contain sample from patients with no cancer (nc). The remaining lanes were renumbered in **Fig 3** starting from the NSCLC patients as typed on the figures here. Samples from early stage lung cancer patients are in lanes 1-5 (histotype is denoted in black in the key), and samples from late stage lung cancer patients are in lanes 6-10 (histotype denoted in red). Purified CFH is in the last lane.The molecular mass of the protein markers are: 250, 130, 100, 70, 55, 35, and 25 kDa. The Mr of CFH is 160 kDa. The relevant section of the blots are shown in the final figure.

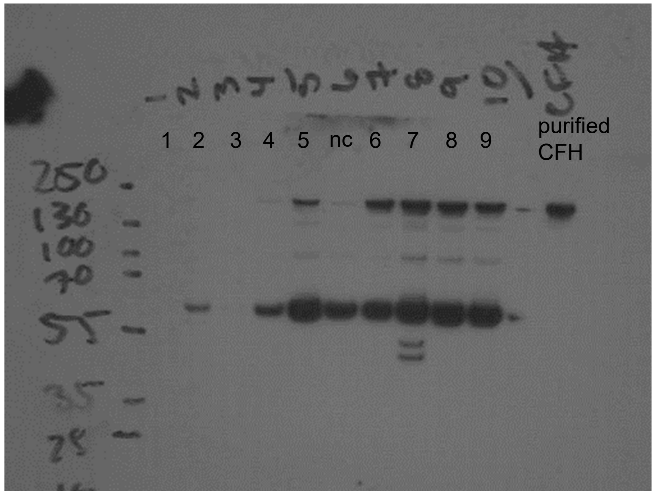

Key
1 – Adenocarcinoma stage IA
2 – Adenocarcinoma stage IB
3 – Squamous cell carcinoma stage IA
4 – Adenocarcinoma stage IA
5 – Adenocarcinoma stage IA
Control with no cancer (nc)
6 – Small cell lung cancer stage IIIA/B
7 – Adenocarcinoma stage IIIA
8 – Undifferentiated NSCLC stage IIIB
9 – Small cell lung cancer stage III
CFH Protein, human, 20 ng (Complement Technology, Inc.)

Creation of S2 Fig: CFH in ultracentrifugation pellet of additional lung cancer patients. Extracellular vesicles were isolated from patient plasma by ultracentrifugation as described in the Methods. The samples were run in a gel and western blotted. However, unlike the western blots of **Fig 3**, here the probes were human GT103 as primary antibody and an anti-human-HRP secondary antibody-conjugate. The Mr of CFH is 160 kDa. The heavy band running at approximately 60 kDa in most lanes may be carryover IgG heavy chain reacting with the secondary antibody. It is absent in the **Fig 3** western blots that were probed with murinized GT103 and an anti-mouse-HRP conjugate secondary antibody conjugate in order to avoid this problem. As renumbered, samples from early stage lung cancer patients are in lanes 1-5 (histotype is denoted in black in the key), a sample from a control patient with no cancer (nc) is in the next lane, and samples from late stage lung cancer patients are in lanes 6-9 (histotype denoted in red). Purified CFH is in the last lane. The molecular masses of the protein markers are: 250, 130, 100, 70, 55, 35, and 25 kDa. The section of the blot containing CFH is shown in the final figure.
