# Supplementary material for: Complement factor H protects tumor cell-derived exosomes from complement-dependent lysis and phagocytosis
Source: PLoS One. 2021 Jun 16;16(6):e0252577. doi: 10.1371/journal.pone.0252577 (PMC8208531; doi:10.1371/journal.pone.0252577)
Supplement: S1 Fig — PBMCs isolated from the blood of a normal volunteer and the NCI-460 lung tumor cell line were each cultured in exosome free medium. EVs were isolated from the conditioned media using the Total Exosome Isolation Kit (Invitrogen 4478359) and conjugated to anti-CD63 beads (Invitrogen 10606D). (A) Antibody binding to exosomes. Binding of GT103 or control IgG to the exosome-bead conjugates was measured by flow cytometry. (B) Antibody-mediated lysis of exosomes. Lysis of exosomes in exosome-bead conjugates in the presence of GT103 or IgG and NHS as a source of complement was measured by flow cytometry in a calcein release assay in which loss of label is indicative of lysis. In both experiments, reactions were run in duplicate. Data are represented as mean +/- SD; significance was assessed using Student’s t-test. *P<0.05; **P>0.05. (PDF) [file pone.0252577.s003.pdf]

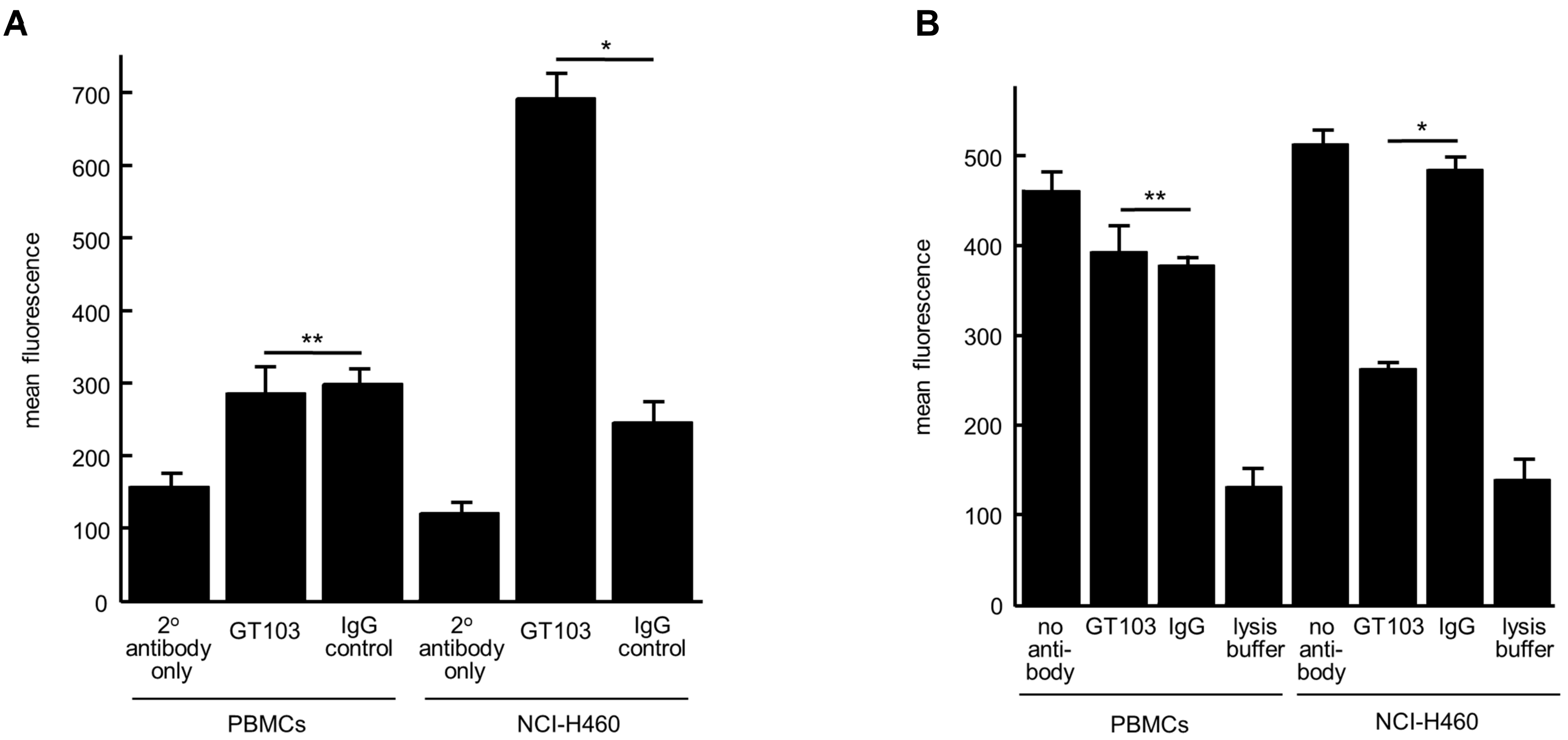

S1 Fig

GT103 vs. control antibody binding and lysis of exosomes from PBMCs and NHI-H460 lung tumor cells

PBMCs isolated from the blood of a normal volunteer and the NCI-460 lung tumor cell line were each cultured in exosome free medium. EVs were isolated from the conditioned media using the Total Exosome Isolation Kit (Invitrogen 4478359) and conjugated to anti-CD63 beads (Invitrogen 10606D). **(A)** Antibody binding to exosomes. Binding of GT103 or control IgG to the exosome-bead conjugates was measured by flow cytometry. **(B)** Antibody-mediated lysis of exosomes. Lysis of exosomes in exosome-bead conjugates in the presence of GT103 or IgG and NHS as a source of complement was measured by flow cytometry in a calcein release assay in which loss of label is indicative of lysis. In both experiments, reactions were run in duplicate. Data are represented as mean +/- SD; significance was assessed using Student's t-test. *P<0.05; **P>0.05.
