# Supplementary material for: Complement factor H protects tumor cell-derived exosomes from complement-dependent lysis and phagocytosis
Source: PLoS One. 2021 Jun 16;16(6):e0252577. doi: 10.1371/journal.pone.0252577 (PMC8208531; doi:10.1371/journal.pone.0252577)
Supplement: S2 Fig — EVs were isolated from patient plasma by ultracentrifugation and subjected to western blot analysis for CFH. The blot was probed with human GT103 as primary antibody followed by an anti-human-HRP secondary antibody-conjugate. Samples from early stage lung cancer patients are in lanes labeled 1–5 (histotype denoted in black), a sample from a control patient with no cancer (nc) is in the next lane, and samples from late stage lung cancer patients are in lanes labeled 6–9 (histotype denoted in red). Note lane 5 contains CFH-positive EVs from an early stage lung cancer patient mentioned in the text. (PDF) [file pone.0252577.s004.pdf]

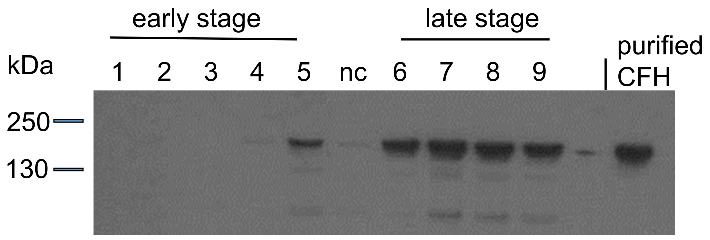

Key

1 – Adenocarcinoma stage IA
2 – Adenocarcinoma stage IB
3 – Squamous cell carcinoma stage IA
4 – Adenocarcinoma stage IA
5 – Adenocarcinoma stage IA
nc – Control with no cancer
6 – Small cell lung cancer stage IIIA/B
7 – Adenocarcinoma stage IIIA
8 – Undifferentiated NSCLC stage IIIB
9 – Small cell lung cancer stage III
CFH Protein, human, 20 ng (Complement Technology, Inc.)

S2 Fig

GT103 western blot film of extracellular vesicles from 9 additional lung cancer patients

EVs were isolated from patient plasma by ultracentrifugation and subjected to western blot analysis for CFH. The blot was probed with human GT103 as primary antibody followed by an anti-human-HRP secondary antibody-conjugate. Samples from early stage lung cancer patients are in lanes labeled 1-5 (histotype denoted in black); a sample from a control patient with no cancer (nc) is in the next lane, and samples from late stage lung cancer patients are in lanes labeled 6-9 (histotype denoted in red). Note lane 5 contains CFH-positive EVs from an early stage lung cancer patient mentioned in the text.
