# Supplementary material for: Complement factor H protects tumor cell-derived exosomes from complement-dependent lysis and phagocytosis
Source: PLoS One. 2021 Jun 16;16(6):e0252577. doi: 10.1371/journal.pone.0252577 (PMC8208531; doi:10.1371/journal.pone.0252577)
Supplement: S3 Fig — A549 human lung cancer cells were incubated with no antibody, GT103, or IgG negative control in the presence of intact normal human serum (NHS), or serum depleted (Dpl) of Factor B (FB), C1q, or C4. After 24 hrs, lysis was measured by lactose dehydrogenase release using the CytoTox 96® Non-Radioactive Cytotoxicity Assay (Promega, Madison, WI) according to the manufacturer’s instructions, and expressed as percent cytotoxicity. In addition, cells incubated with heat inactivated NHS (HI-NHS) were included as a control for spontaneous lysis of cells occurring in serum with no complement or antibody; cells incubated with NHS were included as a control for spontaneous CDC in serum with no antibody. All reactions were run in triplicate. Data are represented as mean +/- SD; significance was assessed using Student’s t-test. *P<0.05. (PDF) [file pone.0252577.s005.pdf]

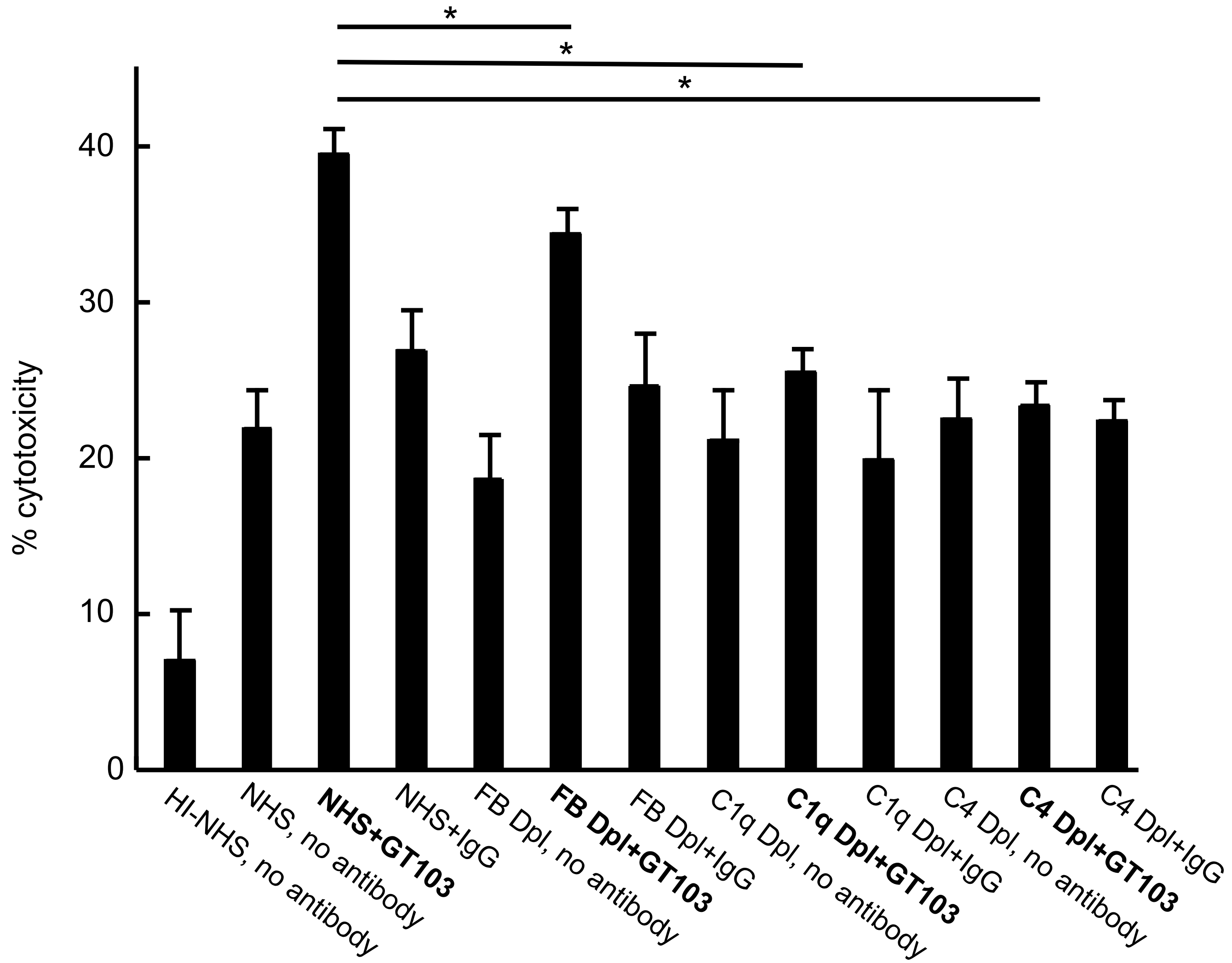

S3 Fig

Complement dependent cytotoxicity (CDC) in the presence of normal or complement depleted sera

A549 human lung cancer cells were incubated with no antibody, GT103, or IgG negative control in the presence of intact normal human serum (NHS), or serum depleted (Dpl) of Factor B (FB), C1q, or C4. After 24 hrs, lysis was measured by lactose dehydrogenase release using the CytoTox 96® Non-Radioactive Cytotoxicity Assay (Promega, Madison, WI) according to the manufacturer's instructions, and expressed as percent cytotoxicity. In addition, cells incubated with heat inactivated NHS (HI-NHS) were included as a control for spontaneous lysis of cells occurring in serum with no complement or antibody; cells incubated with NHS were included as a control for spontaneous CDC in serum with no antibody. All reactions were run in triplicate. Data are represented as mean +/- SD; significance was assessed using Student's t-test. *P<0.05.
